# HIV-1 controllers exhibit an enhanced antiretroviral innate state characterised by overexpression of p21 and MCPIP1 and silencing of ERVK-6 RNA expression

**DOI:** 10.1590/0074-02760240071

**Published:** 2024-09-16

**Authors:** Suwellen Sardinha Dias de Azevedo, Marcelo Ribeiro-Alves, Fernanda Heloise Côrtes, Edson Delatorre, Brenda Hoagland, Larissa M Villela, Beatriz Grinsztejn, Valdilea Gonçalvez Veloso, Mariza G Morgado, Thiago Moreno L Souza, Gonzalo Bello

**Affiliations:** 1 Fundação Oswaldo Cruz-Fiocruz, Instituto Oswaldo Cruz, Laboratório de AIDS & Imunologia Molecular, Rio de Janeiro, RJ, Brasil; 2 Fundação Oswaldo Cruz-Fiocruz, Instituto Nacional de Infectologia Evandro Chagas, Laboratório de Pesquisa Clínica em DST/AIDS, Rio de Janeiro, RJ, Brasil; 3 Universidade Federal do Espírito Santo, Centro de Ciências da Saúde, Departamento de Patologia, Laboratório de Genômica e Ecologia Viral, Vitória, ES, Brasil; 4 Fundação Oswaldo Cruz-Fiocruz, Instituto Oswaldo Cruz, Laboratório de Imunofarmacologia, Rio de Janeiro, RJ, Brasil; 5 Fundação Oswaldo Cruz-Fiocruz, Instituto Nacional de Ciência e Tecnologia de Inovação em Doenças de Populações Negligenciadas, Rio de Janeiro, RJ, Brasil; 6 Fundação Oswaldo Cruz-Fiocruz, Instituto Oswaldo Cruz, Laboratório de Arbovírus e Vírus Hemorrágicos, Rio de Janeiro, RJ, Brasil

**Keywords:** HIV-1, ERVK-6, elite controllers, viremic controllers, restriction factors

## Abstract

**BACKGROUND:**

Human immunodeficiency virus (HIV)-1 infection can activate the expression of human endogenous retroviruses (HERVs), particularly HERV-K (HML-2). HIV controllers (HICs) are rare people living with HIV (PLWHs) who naturally control HIV-1 replication and overexpress some cellular restriction factors that negatively regulate the LTR-driven transcription of HIV-1 proviruses.

**OBJECTIVES:**

To understand the ability of HICs to control the expression of endogenous retroviruses.

**METHODS:**

We measured endogenous retrovirus type K6 (ERVK-6) RNA expression in peripheral blood mononuclear cells (PBMCs) of HICs (n = 23), antiretroviral (ART)-suppressed subjects (n = 8), and HIV-1-negative (NEG) individuals (n = 10) and correlated the transcript expression of ERVK-6 with multiple HIV-1 cellular restriction factors.

**FINDINGS:**

Our study revealed that ERVK-6 RNA expression in PBMCs from HICs was significantly downregulated compared with that in both the ART and NEG control groups. Moreover, we detected that ERVK-6 RNA levels in PBMCs across all groups were negatively correlated with the expression levels of p21 and MCPIP1, two cellular restriction factors that limit the activation of macrophages and T cells by downregulating the activity of NF-kB.

**MAIN CONCLUSIONS:**

These findings support the hypothesis that HICs activate innate antiviral mechanisms that may simultaneously downregulate the transcription of both exogenous (HIV-1) and endogenous (ERVK-6) retroviruses. Future studies with larger cohorts should be performed to confirm this hypothesis and to explore the role of p21 and MCPIP1 in regulating HERV-K expression in physiological and pathological conditions.

Human endogenous retroviruses (HERVs) are remnants of ancient viral infections integrated into the human genome throughout evolutionary history. These genetic elements are estimated to comprise nearly 8% of the human genome and are passed from generation to generation.^1^ Although most HERVs are now functionally inactive due to mutations and deletions, some have been implicated in disease processes, such as autoimmune and neurodegenerative disorders, several types of cancer, and immune dysregulation and coagulopathy in critical coronavirus disease 2019 (COVID-19).^2^ ^-^ ^9^

HERV-K represents the latest classified family, encompassing the most recently integrated HERV groups within the human genome.^10^ HERV-Ks can be further divided into ten subfamilies (HML 1 - 10), with some retaining their structural integrity and ability to generate viral proteins such as HERV-K (HML-2).^11^ Interestingly, previous studies revealed that human immunodeficiency virus (HIV)-1 can activate HERV-K (HML-2) mRNA and protein expression *in vitro*.^12^ ^,^ ^13^ ^,^ ^14^ ^,^ ^15^ Moreover, untreated people living with HIV (PLWHs) display higher levels of HERV-K RNA in peripheral blood mononuclear cells (PBMCs)^4^ ^,^ ^13^ ^,^ ^16^ or plasma^17^ ^,^ ^18^ ^,^ ^19^ than antiretroviral therapy (ART)-treated individuals and/or HIV-uninfected healthy subjects. One study demonstrated that HIV-1 Tat plays an important role in activating the expression of HERV-K (HML-2).^20^ HIV-1 infection may also enhance HERV-K (HML-2) transcription in both infected and uninfected cells,^4^ probably by indirect mechanisms influencing immune activation and inflammation^21^ and/or the cellular epigenetic environment.^22^

These findings suggest that LTR-directed transcription of both HERV-K and HIV-1 proviruses could be regulated by common viral and cellular transcription factors. We previously observed that the expression levels of two multifunctional cellular proteins, cyclin-dependent kinase (CDK) inhibitor 1A (CDKN1A/p21) and monocyte chemotactic protein-induced protein 1 (MCPIP1), were significantly elevated in a group of rare PLWHs who naturally control plasma viremia without ART, called HIV controllers (HICs), compared with ART-suppressed and HIV-1-negative individuals.^23^ Interestingly, p21 and MCPIP1 are critical to maintaining immune-system homeostasis and contribute to limiting the activation of macrophages and T cells by downregulating NF-kB activity,^24^ ^,^ ^25^ ^,^ ^26^ ^,^ ^27^ ^,^ ^28^ ^,^ ^29^ a transcription factor that stimulates the LTR-driven activation of both HIV-1 and HERV-K proviruses. Thus, HICs overexpress cellular restriction factors that may negatively regulate the transcription of HERV-K. However, the precise relationship between the natural control of HIV replication and the expression of HERVs remains unclear.

To test the hypothesis that cellular restriction factors overexpressed in HIC may also negatively regulate HERVs, we performed an exploratory analysis focused on the transcriptional activity of endogenous retrovirus type K6 (ERVK-6) proviral locus that belongs to the HERV-K (HML-2) subfamily and has been reported to be activated in cells persistently infected by HIV.^14^ We measured the ERVK-6 RNA expression levels (envelope protein fragment, also called HERV-K [HML-2] or HERV-K_7p22.1) in PBMCs of HICs (n = 23), HIV-1 ART-suppressed subjects (n = 10) and HIV-1-negative individuals (n = 8), and we also correlated the transcript expression levels of ERVK-6 with multiple HIV-restriction factors.

## SUBJECTS AND METHODS

*Study subjects* - We analysed a cohort of 23 HICs followed up at the Instituto Nacional de Infectologia Evandro Chagas (INI) in Rio de Janeiro, Brazil and provided written informed consent documents approved by the INI Institutional Review Board (Addendum 049/2010) and the Brazilian National Human Research Ethics Committee (CONEP 14430/2011). The procedures followed were in accordance with the Helsinki Declaration of 1975, as revised in 1983.

All HICs maintained an RNA VL of < 2000 copies/mL without antiretroviral therapy for at least five years and were subdivided into two subgroups: elite controllers (EC, n = 13) in whom most (≥ 70%) plasma VL determinations were below the limit of detection (LOD) and viremic controllers (VC, n = 10) in whom most (≥ 70%) VL determinations were > LOD and < 2000 copies/mL. The limit of detection of plasma VL determinations varied over the follow-up period according to the Brazilian Ministry of Health guidelines, with methodologies being updated over time to improve sensitivity: Nuclisens HIV-1 RNA QT assay (Organon Teknika, Durham, NC, USA, limit of detection: 80 copies/mL) from 1999 to 2007; the Versant HIV-1 3.0 RNA assay (bDNA 3.0, Siemens, Tarrytown, New York, NY, USA, limit of detection: 50 copies/mL) from 2007 to 2013; and the Abbott RealTime HIV-1 assay (Abbott Laboratories, Wiesbaden, Germany, limit of detection: 40 copies/mL) from 2013 to present. Previous studies detailed these subjects’ virological and immunological characteristics.^23^ ^,^ ^30^ ^,^ ^31^ ART-suppressed subjects (ART, n = 8) and healthy HIV-1-uninfected subjects (NEG, n = 10) were used as controls.

*mRNA gene-expression analysis* - Total RNA was extracted from 1 × 10^7^ PBMCs using an RNeasy mini kit (Qiagen, North Rhine-Westphalia, Hilden, Germany) in which buffer RLT was supplemented with β-mercaptoethanol and displaced on-column DNase treatment using a Qiagen RNase-Free DNase Set (Qiagen, North Rhine-Westphalia, Hilden, Germany) according to the manufacturer’s instructions. Total RNA yield and quality were determined using NanoDrop^®^ 8000 spectrophotometer and Agilent^®^ (Santa Clara, CA, USA) 2100 Bioanalyzer. Only samples with an RNA integrity number (RIN) greater than 8.0 were used for further analysis. Purified RNA (1 μg) was reverse transcribed to cDNA using an RT^2^ First Strand Kit (Qiagen, North Rhine-Westphalia, Hilden, Germany). The cDNA was mixed with RT^2^ SYBR Green/ROX qPCR Master Mix (Qiagen, North Rhine-Westphalia, Hilden, Germany), and the mixture was added to a customised RT^2^ RNA PCR Array (Qiagen, North Rhine-Westphalia, Hilden, Germany) to measure the mRNA expression of ERVK-6 envelope protein, also called HERV-K (HML-2) or HERV-K_7p22.1 (Gene RefSeq #PPH60565A-200/NM_001007236), 13 cellular restriction factors (*CDKN1A/p21*, *ZC3H12A/MCPIP1*, *APOBEC3G*, *IFITM1*, *IFITM2*, *IFITM3*, *SAMHD1*, *Mx1*, *Mx2*, *SERINC3*, *SERINC5*, *SLFN11*, and *Tetherin/Bst2*), and three housekeeping genes (*GAPDH*, *β-actin,* and *RNase-P*), according to the manufacturer’s instructions. Values of the crossing point at the maximum of the second derivative of the four-parameter fitted sigmoid curve second derivative, Cp, were determined for each sample. The efficiency of each amplification reaction was calculated as the ratio between the fluorescence of the cycle of quantification and the fluorescence of the cycle immediately preceding that. Genes used in the normalisation among samples were selected by the geNorm method.^32^ Data were expressed as fold changes in mRNA abundance calculated as the normalised gene expression in any test sample divided by the mean normalised gene expression in the control HIV-1 NEG group.

*Data analyses* - The comparisons of mean log(base 2)-fold changes (log-FC) in mRNA abundance were performed by either nonparametric t tests or one-way analysis of variance (ANOVA) permutation tests (B = 1000 permutations), followed by pairwise comparisons with Holm‒Bonferroni adjustment,^33^ for two or more groups, respectively. The Pearson coefficient was used for correlation analyses. Finally, a multivariate principal component analysis (PCA) was performed for the log-transformed expression data to visualise the distribution of sample individuals according to either their group or their mRNA *ERV-K 6* expression levels (divided into four different quartiles) in two-dimensional (2D) space. The proportion of explained variation was calculated by adding the successive proportions of variation explained to obtain the running total. The contributions (in percentage) of the variables to the principal components were calculated as (var. cos^2^ × 100)/(total cos^2^ of the component), where cos^2^ indicates square cosine or squared coordinates. Accordingly, the contributions (in percentage) of individuals to the principal components were calculated as (ind. cos^2^ × 100)/(total cos^2^ of the component). Ellipses of the quantiles with 66% of the normal distribution adjusted for the individuals in the different interest groups are presented in these new dimensional spaces. A p value ≤ 0.05 was used as the significance level in the analysis. All analyses were performed using R software version 4.1.2^34^ and the packages ‘base’ for descriptive and correlation analyses and ‘FactoMineR’^35^ and ‘factoextra’^36^ for PCA and its graphic representation.

*Availability of data* - The datasets during and/or analysed during the current study are available from the corresponding author upon reasonable request.

## RESULTS

*Epidemiological, clinical and virological characteristics of the studied individuals* - Thirty-one HIV-1-positive (23 HICs and eight ART-suppressed) and 10 HIV-negative individuals were included in this cross-sectional study (Table). The participants had a median age of 46 years [interquartile range (IQR): 40-52 years old]. Most HIV-positive (58%) and HIV-negative (60%) individuals were females, and all individuals displayed CD4^+^ T-cell counts above 500 cells/μL. The HICs were subdivided into EC and VC subgroups based on the history of viral load measurements since the positive diagnosis for HIV-1. The ECs had a median follow-up time of nine years and showed undetectable (< 40-80 copies/mL) viral load at most (≥ 70%) measurements, while the VCs had a median of 10 years of follow-up and showed low-level viremia (81-2000 copies/mL) at most (≥ 70%) visits. The control group of ART-suppressed subjects had a median of 13 years (IQR: 9-22.5) since HIV diagnosis and almost nine years (IQR: 7.25-11) on the use of ART.

**TABLE:** Main epidemiological, clinical, and virological characteristics of individuals

Characteristics	HICs (*n* = 23)		ART (*n* = 8)	NEG (*n* = 10)
	ECs (*n* = 13)	VCs (*n* = 10)		
Sex, no. (%)				
Female	10 (77)	4 (40)	4 (50)	6 (60)
Male	3 (23)	6 (60)	4 (50)	4 (40)
Age (years)*	45 (39-60)	45 (42-49)	47 (38-53)	47 (36-51)
Time since HIV-1 diagnosis (years) *	9 (6-15)	10 (7-15)	13 (9-23)	-
Time since ART started (years)*	NA	NA	9 (7-11)	NA
CD4^+^ T-cell (cells/μL)*	1027 (835-1255)	664 (607-1018)	889 (678-1097)	1043 (784-1581)
CD4/CD8 ratio*	1.33 (1.24-1.61)	0.90 (0.62-1.07)	1.06 (0.73-1.5)	1.69 (1.62-2.00)
Median viremia* (RNA copies/mL)	< 50	719 (512-2242)	< 40	-

ART: antiretroviral therapy; ECs: elite controllers; HICs: human immunodeficiency virus controllers; NEG: negative; VCs: viremic controllers. *At the study point. Interquartile ranges (IQR) are shown in parentheses.

**Fig. 1:**
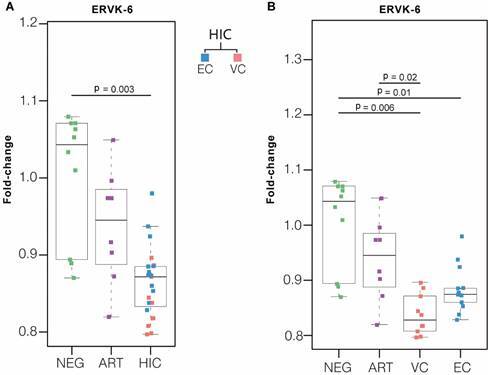
the endogenous retrovirus type K6 (ERVK-6) mRNA levels are downregulated in human immunodeficiency virus controllers (HICs). Boxplots represent the interquartile and sample median (central solid black line) of the relative changes [fold-change values relative to the mean of human immunodeficiency virus (HIV)-1-uninfected (negative - NEG) subjects] of ERVK-6 in HIC (A) and viremic controllers (VCs) and elite controllers (ECs) subgroups (B) compared with NEG and antiretroviral therapy (ART)-suppressed subjects’ (ART) ERVK-6 expression. p-values < 0.05 were considered statistically significant.

*ERVK-6 mRNA levels are downregulated in HICs* - We first evaluated the ERVK-6 transcript levels in the HIC and ART groups compared to the NEG group. We found a significant (p < 0.05) downregulation of *ERVK-6* mRNA in the HIC (0.88 - mean fold-change), VC (0.84 - mean fold-change), and EC subgroups (0.86 - mean fold-change), while no significant differences in *ERVK-6* expression levels were observed between the NEG and ART groups (0.94 - mean fold-change, p = 0.33) (Fig. 1). In comparison to the ART group, we found a significant downregulation of *ERVK-6* mRNA in VCs (0.89-mean fold-change, p = 0.02) and a lower, but not significant, expression in ECs (0.94-mean fold-change, p = 0.70) and HICs (0.92-mean fold-change, p = 0.08) (Fig. 1). No significant differences in *ERVK-6* expression levels were observed between the EC and VC subgroups (p = 0.08) (Fig. 1).

**Fig. 2:**
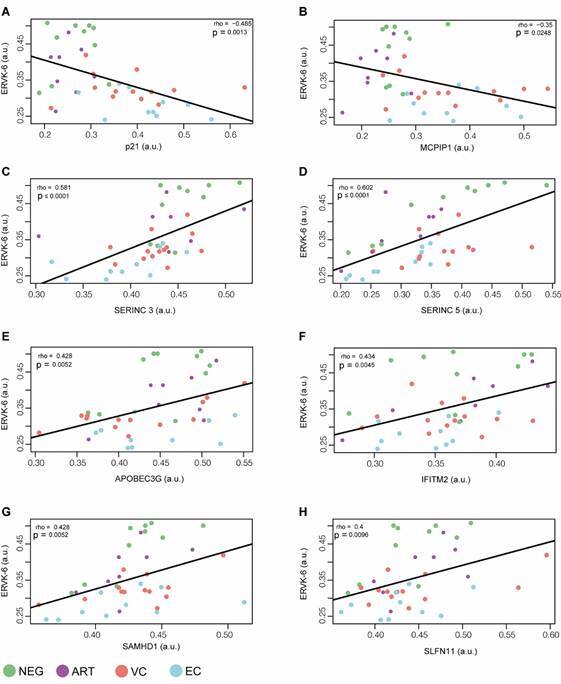
correlation between mRNA levels of endogenous retrovirus type K6 (ERVK-6) and some restriction factors (RFs): p21 (A); MCPIP1 (B); SERINC3 (C); SERINC5 (D); APOBEC3G (E); IFITM2 (F); SAMHD1 (G), and SLFN11 (H) in peripheral blood mononuclear cells (PBMCs) from human immunodeficiency virus controllers (HICs) [viremic controllers (VCs) and elite controllers (ECs)] and control groups [negative (NEG) and antiretroviral therapy (ART)]. The points’ colours indicate the patient group, according to the legend. Correlation coefficients (Pearson’s ρ) are shown in each graph’s upper right or left corner. p-values < 0.05 were considered statistically significant.

*ERVK-6 mRNA and cellular restriction factor levels are correlated* - We next assessed whether reduced *ERVK-6* transcripts in HICs could be associated with mRNA expression levels of 13 cellular restriction factors that act against HIV-1. Pearson’s correlation of all groups combined showed that the mRNA expression of *ERVK-6* was negatively correlated with both *p21* (*r* = −0.48; p = 0.0013) and *MCPIP1* (*r* = −0.35; p = 0.0248) (Fig. 2A-B), positively correlated with *SERINC3* (*r* = 0.58; p < 0.0001), *SERINC5* (*r* = 0.60; p < 0.0001), *APOBEC3G* (*r* = 0.43; p = 0.005), *IFITM2* (*r* = 0.43; p = 0.004), *SAMHD1* (*r* = 0.43; p = 0.005), and *SLFN11* (*r* = 0.40; p = 0.009) (Fig. 2C-H), and was not significantly correlated (p > 0.05) with the other five restriction factors (*Mx1*, *Mx2*, *IFITM1, IFITM3*, and *Tetherin*) analysed [Supplementary data (Figure)].

**Fig. 3:**
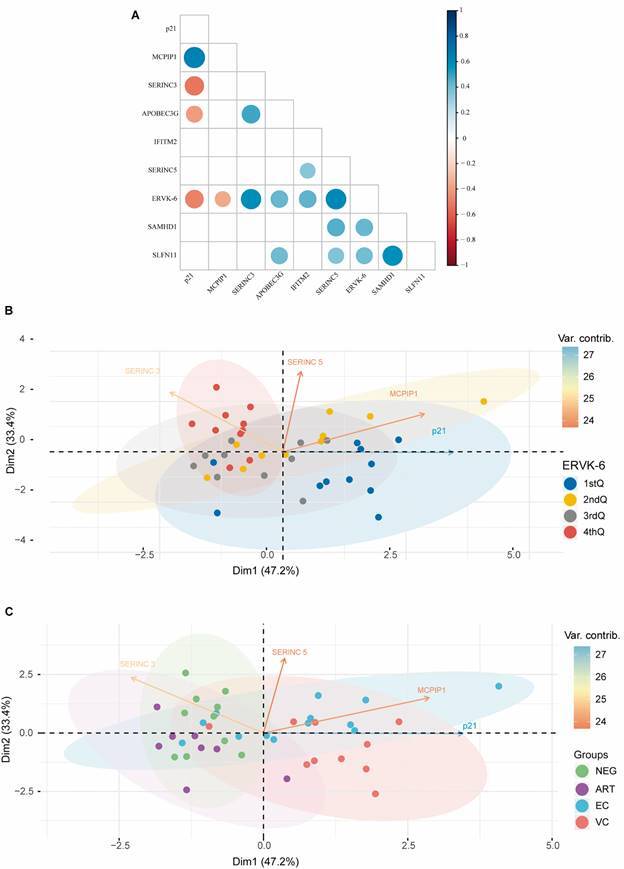
(A) Correlogram representing the matrices of Pearson’s rank-order correlation coefficient (ρ) between restriction factors (RFs) and endogenous retrovirus type K6 (ERVK-6) mRNA expression levels. (B-C) Principal component analysis showing the four principal components of the dataset. In Fig. 3B, each circle represents one sample, and the circle’s colour indicates the mRNA ERVK-6 expression levels (divided into four different quartiles) according to the legend in the right corner. In Fig. 3C, the circles representing each sample are coloured according to groups of the study [negative (NEG), antiretroviral therapy (ART), elite controllers (ECs), and viremic controllers (VCs)], as shown in the legend in the right corner.

We also evaluated the correlation coefficient between restriction factors significantly correlated with *ERVK-6* mRNA expression levels. We detected a significant negative correlation between *p21* and *SERINC3* (*r* = −0.53; p = 0.0004) and *APOBEC3G* (*r* = −0.40; p = 0.009) (Fig. 3A). Significant positive correlations were observed between *SERINC3* and *APOBEC3G* (*r* = 0.49; p = 0.0013) and between *SERINC5* and *SLFN11* (*r* = 0.37; p = 0.018), *SAMHD1* (*r* = 0.45; p = 0.003), and *IFITM2* (*r* = 0.34; p = 0.028). Finally, we also observed positive correlations between *SLFN11* and *APOBEC3G* (*r* = 0.41; p = 0.008) (Fig. 3A).

Multivariate PCA comprising *p21*, *MCPIP1*, *SERINC3*, and *SERINC5* variables is shown in Fig. 3B-C, according to either mRNA *ERVK-6* expression levels divided into four different quartiles (Fig. 3C) or the study group (Fig. 3D). Our results revealed that 80.6% of the total variance in response to the four restriction factors was expressed by two principal components. The first component (Dim1) represented 47.2%, while the second (Dim2) represented 33.4% of the total variance. The variables p21 and MCPIP1 contributed most to the first component (Dim1), while SERINC5 and SERINC3 contributed most to the second component (Dim2).

In general, we observed a strong association between the *ERVK-6* expression quartiles and the HIV-1 control groups (X^2^ = 32.293, df = 9, p = 0.0001772). This association was even more evident between the NEG control group and the quartiles with the highest *ERVK-6* expression (*i.e.*, seven in the fourth quartile, two in the third quartile, one in the second quartile, and none in the first quartile) (X^2^ = 16.056, df = 3, p = 0.001104). The opposite situation, also highly associated, was observed between the VC group and *ERVK-6* expression (seven in the first quartile, one in the second quartile, two in the third quartile, and zero in the fourth quartile) (X^2^ = 13.641, df = 3, p = 0.003437). This association was confirmed when examining the HIC group (ECs + VCs), *i.e.*, 10 in the first quartile, 8 in the second quartile, four in the third quartile, and only one in the fourth quartile (X^2^ = 17.413, df = 3, p = 0.0005812).

The separation between the HICs (ECs + VCs) and the control (NEG + ART) groups occurred almost perfectly along the first component (Dim1), with the first group to the right of the origin (first and fourth quadrants) and the second to the left of the origin (second and third quadrants), except for two ECs and one VC individual, in the third and second quadrants, respectively; two of them had low *ERVK-6* expression (second quartile), and another had very low *ERVK-6* expression (first quartile). The same observation regarding the first component (Dim1) was made for the levels of *ERVK-6* expression, with the individuals with the lowest expression (first and second quartile) being further to the right of the origin (first and fourth quadrants) and those with the highest expression to the left of the origin (second and third quadrants). Notably, the individuals with the highest *ERVK-6* expression (fourth quartile) were concentrated in the second quadrant of the PCA, coinciding with the predominance of healthy control individuals (NEG), while the individuals with the lowest *ERVK-6* expression (first quartile) were concentrated in the fourth quadrant of the PCA, coinciding with the predominance of VCs.

## DISCUSSION

Previously published studies revealed that HERV-K (HML-2) RNA expression in PBMCs from untreated viremic PLWHs was higher than that in ART-treated PLWHs and/or HIV-uninfected healthy subjects.^4^ ^,^ ^13^ ^,^ ^16^ However, none of these studies analysed HERV-K (HML-2) RNA expression in HICs. Our study reveals that *ERVK-6* (HML-2) RNA expression in PBMCs from HICs, particularly the VC subgroup, was significantly downregulated compared with both ART-treated PLWHs and/or HIV-uninfected healthy control groups.

Our analyses revealed that *ERVK-6* RNA level in our group of ART-treated PLWHs with undetectable viremia was comparable to those detected in HIV-uninfected healthy, consistent with the notion that successful ART may reduce the expression level of HERVs in HIV-infected individuals.^18^ Other studies, by contrast, described the persistently elevated expression of some HERVs, including HERV-K (HML-2), in PBMCs of HIV patients under ART compared to healthy controls.^4^ ^,^ ^37^ The contradictory results could be explained by varying levels of residual replication and duration of therapy among ART-treated PLWHs, or by the influence of factors other than HIV replication on the expression of HERVs. Indeed, we would expect HICs to exhibit slightly higher expression levels than both control groups in our study because HICs usually displayed higher residual viremia than ART-treated PLWHs.^38^ ^,^ ^39^ Thus, the downregulation of *ERVK-6* RNA levels observed in our group of HICs cannot be attributed solely to the extremely low viral load.

We hypothesise that HICs may activate some antiviral mechanisms that control the expression of both exogenous and endogenous retroviruses. Consistent with this notion, we detected that *ERVK-6* RNA levels in PBMCs were negatively correlated with the expression levels of two cellular restriction factors, namely, *p21* and *MCPIP1*. These proteins can block HIV-1 replication in macrophages and CD4^+^ T cells^40^ ^,^ ^41^ ^,^ ^42^ ^,^ ^43^ ^,^ ^44^ and further limit aberrant immune activation.^24^ ^,^ ^45^ ^,^ ^46^ Interestingly, p21 and MCPIP1 limit the activation of macrophages and T cells by downregulating the activity of NF-κB,^24^ ^,^ ^25^ ^,^ ^26^ ^,^ ^27^ ^,^ ^28^ ^,^ ^29^ a transcription factor that stimulates the LTR-driven transcription of HIV-1 and HERV-K proviruses.^47^ ^,^ ^48^ In a previous study, we showed that *MCPIP1* and *p21* mRNA and protein expression levels were upregulated in PBMCs from our HIC cohort.^23^ Therefore, we suggest that HICs activate a homeostatic anti-inflammatory response that comprises antiviral factors, such as p21 and MCPIP1, to prevent excessive immune activation driven by residual HIV-1 replication. This negative homeostatic response inhibits the NF-κB pathway, which may in turn reduce the efficiency of the LTR-driven transcription of both HIV-1 and HERV-K proviruses.

Interestingly, we detected that *ERVK-6* RNA expression in our cohort was positively correlated with two cellular restriction factors, *SERINC3* and *SERINC5*, that may upregulate HERV-K (HML-2) RNA levels by activating the NF-κB pathway. SERINC3/5 were initially identified as cell restriction factors that can potently suppress HIV-1 infectivity by incorporating into budding viral particles and impairing subsequent virion fusion and infection of new target cells.^49^ ^,^ ^50^ A recent study revealed that SERINC3/5 exhibited additional antiviral activities by forming a signalling complex with the mitochondrial antiviral signalling protein (MAVS) and TRAF6 at the mitochondria that increased the phosphorylation and activation of IRF3 and IκBα, thus cooperatively enhancing the NF-κB inflammatory pathway and type I IFN (IFN-I) production.^51^ Moreover, we detected a significant negative correlation between the expression of *p21* and *SERINC3*, suggesting that these restriction factors may display antagonist transcriptional regulation and may be part of the mechanism that controls the repression and activation of HERVs.

Our findings also revealed that the expression of some ISGs analysed here (*APOBEC3G*, *SLFN11*, and *SAMDH1*) was positively correlated with the expression of both *ERVK-6* and *SERINC3/5*. Cytosolic HERV-K dsRNA/cDNA may activate different nucleic acid sensors involved in innate immunity, such as retinoic acid-inducible gene-I-like receptors (RIG-I and MDA5)^52^ ^,^ ^53^ ^,^ ^54^ and cGMP-AMP synthase (cGAS).^55^ Activation of these innate immune sensors leads to phosphorylation and activation of TANK-binding kinase 1 (TBK1) and IκB kinase-ϵ (IKKϵ) kinases, which in turn activate the transcription factors IRF3 and NF-κB, ultimately leading to upregulation of type I IFNs, ISGs, and other proinflammatory cytokines. Interestingly, SAMHD1 and SLFN11 belong to a subset of ISGs that may be directly induced by IRF3,^56^ ^,^ ^57^ and APOBEC3G may be positively modulated by the NF-κB pathway.^58^ We propose that increased levels of SERINC3/5 and cytosolic HERV-K RNA/DNA may directly upregulate some ISGs by triggering the activation of IRF-3 and NF-κB, independent of the engagement of the type I IFN-JAK-STAT pathway.

Previous studies described that HICs have more robust cellular and antibody responses against HERV-K than ART-suppressed, viremic no controllers and uninfected subjects.^59^ ^,^ ^60^ The generation and maintenance of strong HERV-specific cellular and antibody responses in HICs seem inconsistent with the very low ERVK-6 mRNA expression detected here in PBMCs. Some findings, however, may explain these apparently conflicting results. Previous studies conducted in our and other cohorts of HICs showed that HIV-1 continues to replicate and evolve despite undetectable or extremely low levels of viremia,^30^ ^,^ ^61^ ^-^ ^66^ and HIV-1 replication in HICs seems to occur mainly in the lymph nodes.^66^ Thus, HERV-K expression in HICs may be mostly restricted to HIV-infected cells residing in lymph nodes, and the expression of both HERV-K and HIV-1 antigens at those sites may stimulate the continuous generation of HERV-specific cellular and antibody responses. In contrast, most PBMCs and HIV-infected cells in the periphery display an antiviral transcriptional signature that limits the activation of both exogenous and endogenous retroviruses.

Persistent overexpression of IFN-α/β and ISGs is a hallmark of chronic HIV-1 infection and is associated with detrimental immune activation, bystander CD4^+^ T-cell apoptosis, and progressive disease.^67^ ^,^ ^68^ Some data suggest that augmented HERV expression may play an active role in exacerbating and perpetuating chronic inflammation in some autoimmune diseases via type I IFN- and MAVS-positive signalling feedback loops.^69^ On the other hand, HERV-K (HML-2) knockdown significantly downregulated genes containing interferon-stimulated response elements (ISREs) in their promoters in basal and IFN-γ-challenged macrophages and had implications for the paracrine activation of nearby cells following macrophage activation.^21^ Moreover, repression of endogenous retrovirus *in vivo* alleviates tissue aging and, to some extent, organismal aging by attenuating innate immune responses.^54^ Thus, repression of HERV-K expression in HICs may contribute to attenuating the activation of innate immune responses, preventing chronic overexpression of IFN-α/β/ISGs and preserving immune homeostasis in those subjects.

Previous studies from our cohort and other groups showed that HICs displayed elevated levels of some key plasma inflammatory markers, such as sCD14, IP-10, IL-18, and D-dimer, and higher CD8^+^ T-cell activation concerning HIV-negative individuals.^31^ ^,^ ^70^ ^-^ ^76^ Moreover, VCs displayed higher levels of immune activation and residual inflammation than ECs. The proinflammatory profile observed in HICs, however, is different from the aberrant inflammation observed in untreated no controllers.^77^ ^,^ ^78^ Indeed, gene expression analysis of PBMCs and purified T cells from HICs showed that these individuals, compared with ART-treated individuals, have lower expression of several inflammatory genes, such as IL1A/B, IL-6, CXCL5, CXCL13, and CXCL1, but maintain upregulation of genes associated with cytotoxicity and the T-cell response.^79^ We suggest that the low levels of ERVK-6 detected here in HICs may be linked to the upregulation of an anti-inflammatory response that limits aberrant inflammation, and such homeostatic mechanisms would be more critical for VCs due to the higher levels of chronic HIV-1 antigenic stimulation compared with ECs.

A recent study, made available in preprint format, that compared the (retro)transcriptome of ECs, PLWH-on-ART, viremic progressors and healthy controls supports a quite different scenario.^80^ According to this study, ECs display elevated levels of specific ISGs and transposable elements (TEs) located upstream of ISGs for control groups. The authors propose a model in which ECs upregulate TEs serving as promoters or enhancers for ISGs that inhibit HIV replication. This is markedly different from our model of natural control, but several factors make direct comparisons between studies challenging. First, different HERV proviruses may respond differently to HIV infection.^12^ ^,^ ^37^ ^,^ ^81^ ^,^ ^82^ Second, the referenced study focused on analysing HERVs expression using previously published RNAseq data from activated CD4^+^ T-cells, differing greatly from our study of *ex vivo* (unstimulated) PBMCs. Third, the preprint study described different (retro)transcriptomic profiles among different ECs, reinforcing the heterogeneity of resistance mechanisms among ECs cohorts.

Our study has two main limitations. The first limitation is the small sample size. Our study was exploratory and included a relatively small number of HICs, so future studies with larger cohorts of ECs and VCs are needed to confirm the findings described here. The second limitation is that we do not provide a comprehensive overview of the expression profile of all HERV-K (HML-2) loci. Our exploratory analysis was centred on the transcriptional activity of ERVK-6 because this proviral locus was shown to be activated in persistently HIV-infected cells.^14^ However, different HERV-K (HML-2) loci could be differentially regulated by either HIV-1 infection or proinflammatory stimuli, and the ERVK-6 provirus represents a small percentage (< 0.2%) of the total HERV-K HML-2 expression under physiological conditions.^12^ ^,^ ^21^ Thus, future investigations exploring the expression of different HERV-K (HML-2) loci are essential to understanding the ability of HICs to downregulate the expression of this human endogenous retrovirus group-K subgroup.

In summary, our findings revealed that ERVK-6 mRNA levels in PBMCs of HICs are lower than those seen in aviremic ART-treated subjects and HIV-negative individuals. We found that ERVK-6 mRNA levels were negatively correlated with the expression of some cellular restriction factors (p21 and MCPIP1) and positively correlated with the expression of others (SERINC3/5, APOBEC3G, IFITM2, SAMHD1, and SLFN11). We suggest that reduced expression of ERVK-6 in HICs may result from the upregulation of a distinctive antiviral and anti-inflammatory homoeostatic response that contributes both to control HIV-1 replication and to limit chronic immune activation and inflammation driven by persistent HIV-1 antigenic stimulation. Larger confirmatory studies should be conducted to validate the conclusions of this hypothesis-generating study.
