# Supplementary material for: HIV-1 controllers exhibit an enhanced antiretroviral innate state characterised by overexpression of p21 and MCPIP1 and silencing of ERVK-6 RNA expression
Source: Mem Inst Oswaldo Cruz. 2024 Sep 16;119:e240071. doi: 10.1590/0074-02760240071 (PMC11404982; doi:10.1590/0074-02760240071)
Supplement: Supplementary file 1 [file 1678-8060-mioc-119-e240071-s.pdf]

The following supporting information complements the analysis of this study.

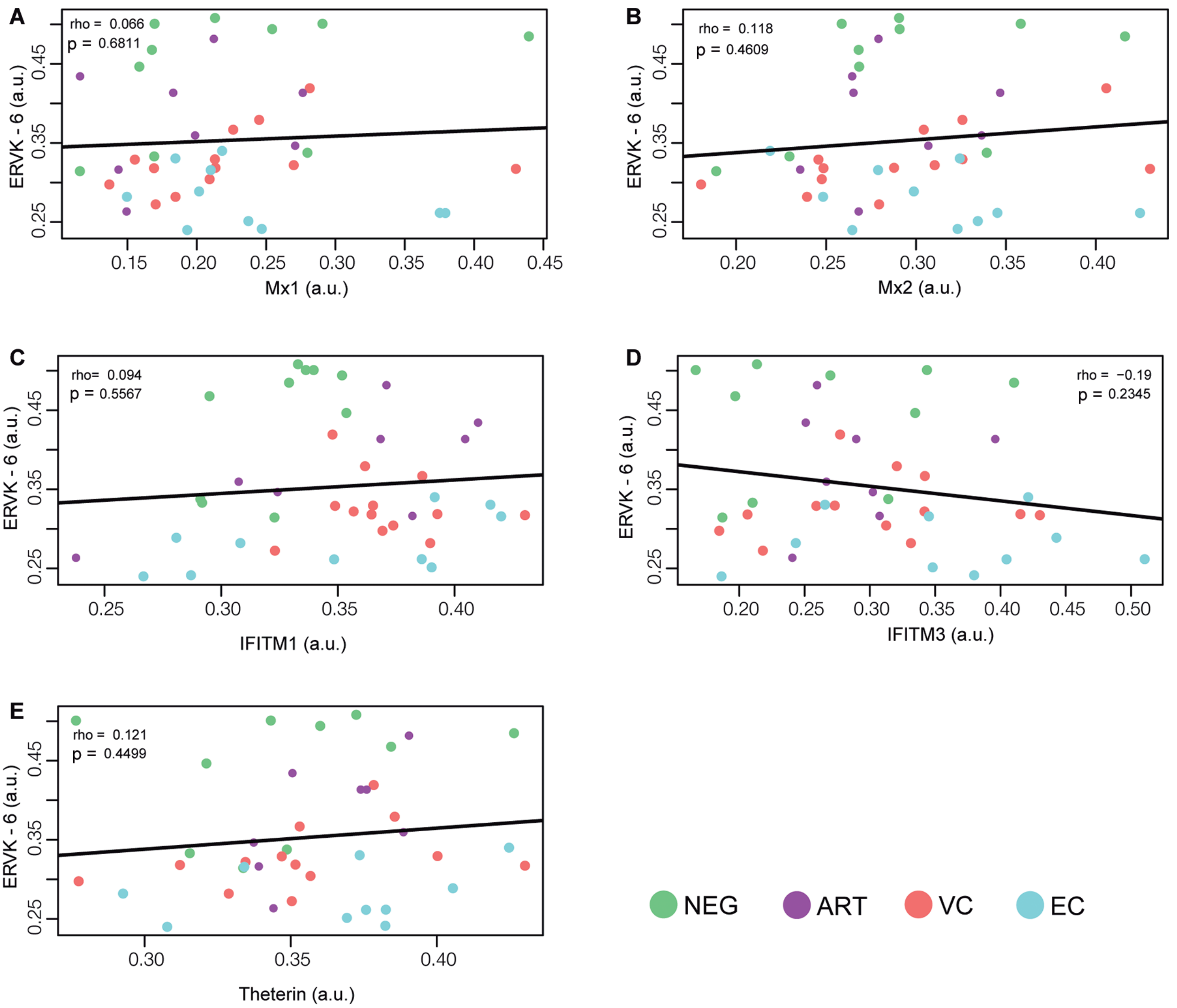

Correlation between restriction factor (RF) mRNA levels in peripheral blood mononuclear cells (PBMCs) from human immunodeficiency virus controllers (HICs) and endogenous retrovirus type K6 (ERVK-6) mRNA expression. The RF-normalised expression correlations were calculated for all groups. The point colours indicate the patient group, according to the legend. Correlation coefficients (Pearson's ρ) are shown in each graph's upper right or left corner. p-values < 0.05 were considered statistically significant.

CC BY
